# Motivational climate, basic psychological needs, intrinsic motivation, and engagement in Chinese students during physical education classes

**DOI:** 10.3389/fpsyg.2026.1913633

**Published:** 2026-10-05

**Authors:** Jun Zhao, Qiang Zhang

**Affiliations:** 1 Graduate School, Harbin Sport University, China; 2 School of Physical Education, Jining University, Qufu, China

**Keywords:** engagement, motivation, motivational climate, physical education, students

## Abstract

**Introduction:**

Although policy initiatives emphasize improving students’ physical health, physical education (PE) classes continue to rely predominantly on traditional teacher-centered approaches, often resulting in insufficient class engagement. Existing research has examined motivational climates, basic psychological needs, intrinsic motivation, and class engagement, yet most studies adopt a single theoretical perspective, offer limited exploration of mediating mechanisms, and provide scarce empirical evidence within the Chinese educational context.

**Methods:**

Data were collected from 1,146 middle school students across five schools in Shandong and Jiangsu provinces using adapted Chinese versions of scales measuring motivational climate, basic psychological needs, intrinsic motivation, and class engagement. Structural equation modeling and bootstrap methods were employed to test the hypotheses.

**Results:**

A mastery climate positively predicted students’ autonomy, competence, and relatedness needs. A performance climate also positively predicted all three basic psychological needs. Autonomy and competence needs positively predicted intrinsic motivation, whereas relatedness need showed no significant predictive effect. Intrinsic motivation positively predicted student engagement. Mediation analyses revealed that basic psychological needs and intrinsic motivation did not form a significant sequential mediating pathway between mastery climate and class engagement. In contrast, a performance climate exerted a positive sequential mediating effect on class engagement through basic psychological needs and intrinsic motivation. Intrinsic motivation mediated the relationships of autonomy and competence needs with class engagement, but did not mediate the link between relatedness need and engagement.

**Discussion:**

To enhance class engagement in PE, it is recommended to optimize the class motivational climates and to deliberately support students’ autonomy and competence needs.

## 1 Introduction

During *the 2026 Two Sessions* in China, the educational philosophy of *“health first”* became a key topic of discussion. *The Government Work Report and the Ministry of Education’s* press conference further emphasized strengthening school physical education (PE), guaranteeing students’ time for physical activity, and tangibly improving the health and fitness levels of youth. Although national policy has elevated school PE to the strategic height of building a *“Healthy China”* and a *“sports power”* (Ministry of Education of the People’s Republic of China, 2025), a paradoxical reality persists in Chinese PE classes, mirroring trends observed internationally (Zamarripa et al., 2021). PE class practice remains largely rooted in a traditional teacher-centered model, prioritizing skill drilling over student-centered participation. This significantly constrains the educational value of PE: First, the dominant, traditional “command-and-control” pedagogical approach positions the teacher as the sole authority, limiting opportunities for student autonomy, creative expression, and meaningful engagement (Mao et al., 2025). In such an environment, students often perceive PE as a compulsory task rather than a source of enjoyment or personal growth (Guo et al., 2022). Second, issues of low class engagement and declining physical fitness remain prominent. Despite policies mandating “one PE class per day” and “two hours of physical activity daily,” student engagement in PE classes remains low, with many displaying diminished interest, reduced effort, or active avoidance (Mao et al., 2025). Against this backdrop, a critical theoretical and practical question emerges: how to implement high-quality PE and health instruction by optimizing the class environment to stimulate active student engagement and maximize the educational efficacy of PE.

Self-Determination Theory (SDT; Ryan and Deci, 2000) and Achievement Goal Theory (AGT; Dweck and Leggett, 1988) provide vital theoretical frameworks for addressing this challenge. The motivational climate in PE, a core concept in AGT, serves as a key mediator linking teaching practice to students’ intrinsic motivation and learning engagement (Hu et al., 2025; Ames, 1992), directly influencing the arousal and maintenance of student motivation and the depth of their involvement. Concurrently, SDT offers a robust framework for understanding how the social environment shapes motivational processes and behavioral outcomes (Deci and Ryan, 2000; Ryan and Deci, 2017). The theory posits that the climate fostered by teachers—whether a mastery-oriented climate emphasizing effort, improvement, and cooperation, or a performance-oriented climate emphasizing ability, comparison, and competition—profoundly impacts students’ satisfaction of their basic psychological needs (Standage et al., 2003; Bartholomew et al., 2011). This need satisfaction, in turn, forms the basis for intrinsic motivation (the inherent desire to participate in the activity itself) and more autonomous forms of extrinsic motivation (Deci and Ryan, 2000). Substantial research confirms that when students perceive autonomy support, opportunities to develop competence, and connection with peers and teachers, they exhibit higher levels of behavioral, emotional, and cognitive engagement in PE (Hospel and Galand, 2016). An integrated perspective combining SDT and AGT can overcome the explanatory limitations of a single theory (Gil-Arias et al., 2020; Soini et al., 2014), clarifying the operative logic of the motivational climate in PE. According to this integrated perspective, class climates that support students’ basic psychological needs are expected to nurture intrinsic motivation and foster deeper learning engagement (Standage et al., 2003). Compared to the high cost and compulsory nature of macro-level fitness promotion policies, optimizing the class motivational climate offers the distinct advantages of being low-cost, highly effective, and non-coercive.

Existing research has explored the relationships between the motivational climate in PE, basic psychological needs, intrinsic motivation, and learning engagement (Hu et al., 2025; Guan et al., 2023; Standage et al., 2005), yielding valuable insights. However, notable gaps remain. First, most studies adopt a single theoretical lens, examining either the effects of mastery climate (MC) and performance climate (PC) based on AGT or the relationships between basic needs and intrinsic motivation based on SDT. This lack of integrative investigation makes it difficult to fully reveal the complete mechanism linking motivational climate, basic psychological needs, intrinsic motivation, and learning engagement. Second, existing research lacks a nuanced exploration of the mediating roles of basic psychological needs and intrinsic motivation between motivational climate and engagement. Furthermore, no consensus has been reached on the differential impacts of different motivational climates on engagement. Third, although SDT has been widely applied in Western PE contexts, empirical evidence from the Chinese educational setting remains relatively limited (Hu et al., 2025). There is insufficient practical exploration of how to enhance student engagement in PE by optimizing the class climate, satisfying basic psychological needs, and activating intrinsic motivation, which hampers the provision of targeted theoretical support and practical guidance for PE teaching reform.

In summary, from a motivational psychology perspective, this study focuses on the intrinsic relationships between the PE class motivational climate, basic psychological needs, intrinsic motivation, and student engagement in PE learning. Adopting an integrated SDT and AGT perspective and drawing on the “social factors—psychological mediators—motivation types—outcomes” motivational sequence model (Vallerand, 1997), this research establishes two core objectives: 1) To construct a theoretical model of “PE class motivational climate → basic psychological needs → intrinsic motivation → PE learning engagement,” propose research hypotheses, and examine the direct effects among variables based on this model; 2) To deeply investigate the mediating effects of the PE class motivational climate and basic psychological need satisfaction on student engagement, systematically revealing the underlying mechanisms.

### 1.1 Hypotheses for direct effects

SDT posits that the support provided by the social environment fosters the development and sustenance of intrinsic motivation by satisfying an individual’s three basic psychological needs: autonomy, competence, and relatedness. AGT further delineates the class motivational climate related to perceptions of ability into an MC and a PC, with the two exerting distinct influences on students’ psychological needs. While extensive research has investigated the link between motivational climate and basic psychological needs in PE, some findings require further validation within the Chinese student population. For instance, research by Chen et al. (2020) indicated that an MC has a significant positive effect on all three psychological needs, whereas the effect of a PC on these needs differs from traditional theoretical expectations. Furthermore, Rodrigues et al. (2020) found that the direction of a PC’s influence on psychological needs is not consistent depending on its source; a PC fostered by teachers often undermines the satisfaction of students’ basic psychological needs. Taken together, theory and evidence consistently support positive mastery-climate predictions, whereas the expected direction of a PC’s associations with need satisfaction remains theoretically contested. Classical AGT and the need-thwarting literature predict that a PC undermines need satisfaction (Dweck and Leggett, 1988; Bartholomew et al., 2011). However, the 2 × 2 achievement-goal framework distinguishes performance-approach from performance-avoidance goal states (Elliot and McGregor, 2001), and recent PE evidence indicates that the implications of a performance climate for need satisfaction are not uniformly negative (Gråstén and Watt, 2017; Huhtiniemi et al., 2022); in the competitive Chinese educational context, a teacher-structured PC may even be experienced as challenging and need-supportive (Chen et al., 2020; Hu et al., 2025). Moreover, the MCPES used in this study captures students’ global perception of a PC and cannot differentiate constructive, structured competition from destructive, evaluative competition, so the competing directional expectations cannot be disentangled *a priori*. We therefore advanced the classic theoretically derived predictions as primary hypotheses and explicitly registered the opposing expectations as competing alternatives. The following hypotheses are proposed regarding motivational climate and basic psychological needs: H1a: A MC is positively associated with students’ autonomy need. H1b: An MC is positively associated with students’ relatedness need. H1c: An MC is positively associated with students’ competence needs. H2a: A PC is negatively associated with students’ autonomy need (primary hypothesis), with a positive association registered as the competing alternative. H2b: A PC is negatively associated with students’ relatedness need (primary hypothesis), with a positive association registered as the competing alternative. H2c: A PC is negatively associated with students’ competence need (primary hypothesis), with a positive association registered as the competing alternative.

Intrinsic motivation, defined as engaging in an activity for its inherent enjoyment and value, constitutes a core motivational type and a key driver for sustaining long-term participation in physical activity (Ryan and Deci, 2000). The motivational sequence model established by Standage et al. (2003) confirms that the satisfaction of basic psychological needs is a crucial precursor to the emergence of intrinsic motivation. Basic Psychological Needs Theory explicitly states that the formation and development of intrinsic motivation depend on the fulfillment of the needs for autonomy, competence, and relatedness (Deci and Ryan, 1985). When students perceive opportunities for choice, skill improvement, and peer support in PE, their level of intrinsic motivation increases significantly. Accordingly, the following are hypothesized: H3a: Autonomy need satisfaction is positively associated with students’ intrinsic motivation. H3b: Relatedness need satisfaction is positively associated with students’ intrinsic motivation. H3c: Competence need satisfaction is positively associated with students’ intrinsic motivation. Learning engagement, representing students’ comprehensive involvement at behavioral, emotional, and cognitive levels in PE classes, is a vital indicator of teaching effectiveness. As the most autonomous form of motivation, intrinsic motivation serves as the core driving force for students’ active participation in PE learning. Stipek (1993) proposed that intrinsic motivation is the fundamental force prompting individuals to engage actively in physical activities. Students with stronger intrinsic motivation are more willing to participate voluntarily in practice, maintain positive affect, and invest cognitive effort in class, ultimately demonstrating higher levels of learning engagement. Therefore, this study hypothesizes: H4: Intrinsic motivation is positively associated with students’ PE learning engagement.

### 1.2 Hypotheses for indirect effects

The formation and enhancement of student learning outcomes in PE is a complex, dynamic process involving multiple interacting factors and pathways. The class context, students’ psychological experiences, and motivational states collectively influence the level of learning engagement. While most current studies examine only the direct link between teaching behaviors and learning outcomes, a deeper exploration of the mediating mechanisms is necessary. To fully reveal the internal pathways through which teachers’ need-supportive instruction influences students’ development of fundamental motor skills and to effectively unpack this “black box,” it is essential to systematically examine the chained mediating effects of core variables. AGT distinguishes between an MC, focused on effort, improvement, and personal growth, and a PC, emphasizing ability comparison and competitive evaluation (Ames, 1992). SDT proposes that the satisfaction of the three basic psychological needs for autonomy, competence, and relatedness is the core prerequisite for the formation and maintenance of intrinsic motivation, with the degree of need satisfaction directly determining the quality of student motivation and level of behavioral engagement (Deci and Ryan, 2000). An integrated perspective of these two theories provides a core framework for analyzing the transmissive mechanism from class climate to learning engagement. Vallerand's (1997) motivational sequence model and empirical research in PE by Standage et al. (2005) confirm that “social context—psychological mediators—motivation types—behavioral consequences” represents a typical pathway for motivational development in PE learning.

Based on this, the following hypotheses are proposed concerning the sequential mediation of motivational climate’s effect on engagement: H5a: Basic psychological need satisfaction and intrinsic motivation sequentially mediate the association between a perceived MC and PE learning engagement. H5b: Basic psychological need satisfaction and intrinsic motivation sequentially mediate the association between a perceived PC and PE learning engagement, with the direction of the indirect effect expected to be negative under the primary AGT prediction and positive under the competing alternative. Furthermore, according to the hierarchical logic of SDT, intrinsic motivation is a key mediator connecting psychological need satisfaction to learning engagement. The satisfaction of each of the three core psychological needs is posited to enhance the level of intrinsic motivation, thereby promoting student engagement in PE learning. Accordingly, the following additional hypotheses are put forward: H6a: Intrinsic motivation mediates the association between autonomy need satisfaction and PE learning engagement. H6b: Intrinsic motivation mediates the association between relatedness need satisfaction and PE learning engagement. H6c: Intrinsic motivation mediates the association between competence need satisfaction and PE learning engagement. Grounded in the aforementioned theoretical rationale and existing research, the theoretical model for this study is constructed, as illustrated in Figure 1.

**Figure 1:**
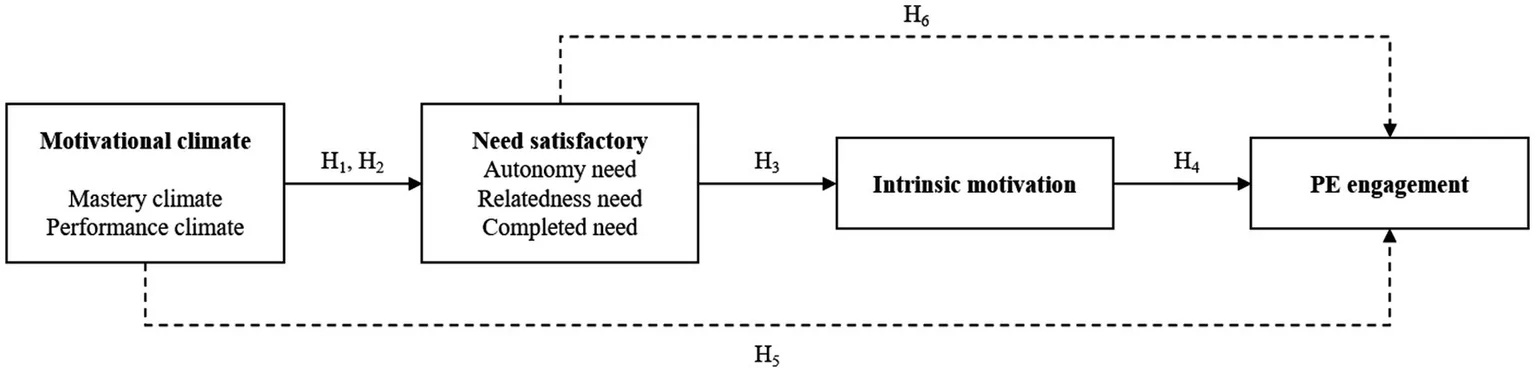
Hypothesized model. Solid lines = direct effects (H₁–H₄); dashed boxes = indirect effects (H₅–H₆) tested via bootstrap mediation.

## 2 Methods

### 2.1 Participants

The sample comprised students in grades 7 to 9 (Junior high school) from five schools located in the coastal provinces of Shandong and Jiangsu in eastern China. Before data collection, the research team obtained approval from school principals, head teachers, and PE teachers. Written informed consent forms, detailing the study’s purpose, procedures, and confidentiality assurances, were distributed to students in the selected classes and their parents or legal guardians. Ultimately, a total of 1,260 students and their guardians provided written informed consent.

### 2.2 Measures

Demographic Variables. Self-reported information, including grade, age, sex, height, and weight, was collected via the questionnaire to characterize the sample.

PE Motivational Climate Scale. The Chinese version of the Motivational Climate in PE Scale (MCPES), originally developed by Soini et al. (2014) and subsequently translated and adapted by Zhang et al. (2024), was employed. Two subscales from the original instrument were used: the MC subscale (e.g., “It is important to do one’s best in PE class”) and the PC subscale (e.g., “It is important to perform better than others”), comprising eight items in total. Responses were recorded on a 5-point Likert scale ranging from 1 (strongly disagree) to 5 (strongly agree), with higher scores indicating a stronger perception of the respective climate. The Cronbach’s alpha for the original scale was 0.87. In the present study, the scale demonstrated good internal consistency with a Cronbach’s alpha of 0.89.

Basic Psychological Needs in PE Scale. The Basic Psychological Needs in PE Scale (BPN-PE), developed by Vlachopoulos et al. (2011) and later translated and revised by Hu et al. (2025), was used. This 12-item scale measures three dimensions: Autonomy Need (4 items, e.g., “The things we do in PE are interesting to me”), Relatedness Need (4 items, e.g., “I get along well with my classmates”), and Competence Need (4 items, e.g., “I feel I can complete even the tasks most students find difficult”). Items were rated on a 7-point Likert scale from 1 (strongly disagree) to 7 (strongly agree). Higher total scores indicate a greater level of basic psychological need satisfaction. The original scale reported a Cronbach’s alpha of 0.88. In this study, the scale showed acceptable internal consistency with a Cronbach’s alpha of 0.80.

PE Learning Motivation Scale. The Chinese version of the Perceived Locus of Causality Revised scale (PLOC-R) for PE, originally adapted by Vlachopoulos et al. (2011) and subsequently translated and revised by Hu et al. (2025), was administered. The 3-item Intrinsic Motivation subscale was utilized (e.g., “I find PE class fun and stimulating”). Responses were given on a 7-point Likert scale (1 = strongly disagree to 7 = strongly agree), with higher scores reflecting a higher level of intrinsic motivation. All items were positively worded. The original subscale had a Cronbach’s alpha of 0.84. In the current sample, it demonstrated good reliability with a Cronbach’s alpha of 0.86.

PE Engagement Scale. The Chinese version of the PE Learning Engagement Scale, originally developed by Agbuga et al. (2015) and later translated and culturally adapted by Zhang (2023), was used. This 13-item scale consists of three subscales: Behavioral Engagement (4 items, e.g., “In PE class, I try as hard as I can to learn”), Emotional Engagement (4 items, e.g., “In PE class, I feel happy”), and Cognitive Engagement (5 items, e.g., “In PE class, I try to connect what I am learning to things I already know”). Participants responded using a 7-point Likert scale ranging from 1 (strongly disagree) to 7 (strongly agree). Higher scores indicate a greater level of engagement in PE. The Cronbach’s alpha for the original scale was 0.91. In this study, the scale showed good internal consistency with a Cronbach’s alpha of 0.83.

### 2.3 Data collection and procedure

This cross-sectional study was conducted in accordance with the Declaration of Helsinki. Ethics approval was obtained from the Academic Committee of Capital University of Physical Education. Written informed consent was obtained from parents or legal guardians, and verbal assent was obtained from participating students before participation. A total of 1,260 questionnaires were distributed online, resulting in a 100% return rate. Following data collection, invalid responses (e.g., patterned responses, missing key information, and excessively short completion times of less than 4 min) were excluded. The final analytical sample consisted of 1,146 students, yielding a valid response rate of 90.95%. The mean age of the participants was 12.55 years (SD = 1.08). The sample included 531 boys. The distribution across grades was 43.96% in grade seven, 33.49% in grade eight, and 21.05% in grade nine.

### 2.4 Data analysis

Data analysis was performed in several stages. Firstly, data screening and preprocessing were conducted. Subsequently, statistical analysis of the key variables—motivational climate, basic psychological needs, intrinsic motivation, and class engagement—was carried out using SPSS 24.0. Preliminary analyses included tests for normality and an assessment of common method bias. Descriptive statistics (means and standard deviations) were calculated for all study variables. Bivariate relationships were examined using Pearson correlation coefficients. The hypothesized relationships and mediating pathways specified in the theoretical model were tested via structural equation modeling (SEM) using AMOS 24.0. Finally, the Bootstrap method (with 5,000 resamples) was employed to test the significance of the indirect effects and verify the robustness of the mediating effects.

## 3 Results

### 3.1 Common method Bias test

Common method bias was assessed using two complementary approaches. First, Harman‘s single-factor test was conducted. Exploratory factor analysis on all items from the four scales extracted nine factors with eigenvalues greater than 1, and the first factor accounted for 20% of the total variance, well below the 40% threshold (Podsakoff et al., 2003). Second, given that Harman’s test is widely regarded as low-power, we further employed the unmeasured latent method construct (ULMC) approach (Podsakoff et al., 2003; Williams et al., 1989). In AMOS, a common latent factor was added to the baseline measurement model, with all observed variables loading onto this orthogonal method factor (variance fixed to 1). The model fit of the method-factor model was then compared with that of the baseline model. Results showed that adding the common method factor did not improve model fit: CFI changed from 0.960 to 0.956 (∆CFI = −0.004), TLI from 0.940 to 0.932 (∆TLI = −0.008), and RMSEA from 0.053 to 0.056 (∆RMSEA = 0.003). All changes were far below the recommended thresholds (∆CFI > 0.10, ∆RMSEA > 0.015 indicate serious bias). Furthermore, the substantive path coefficients and their significance levels remained virtually unchanged after controlling for the method factor. Taken together, these results indicate that common method bias does not pose a serious threat to the validity of our findings.

### 3.2 Measurement model evaluation

Before testing the structural model, we performed confirmatory factor analysis to evaluate the psychometric properties of all measurement instruments. The measurement model included seven latent constructs—mastery climate, performance climate, autonomy need, relatedness need, competence need, intrinsic motivation, and engagement—with their respective observed indicators. Model fit indices indicated acceptable fit: *χ^2^*/*df* = 4.21, GFI = 0.89, NFI = 0.93, IFI = 0.95, TLI = 0.94, CFI = 0.96, RMSEA = 0.05, and SRMR = 0.06. Although GFI fell slightly below the conventional 0.90 threshold, all other indices met or exceeded the recommended criteria (CFI > 0.90, TLI > 0.90, RMSEA < 0.08, SRMR < 0.08), suggesting that the measurement model adequately represented the observed data.

Convergent validity was assessed through standardized factor loadings, composite reliability (CR), and average variance extracted (AVE). All factor loadings exceeded the 0.50 benchmark (range: 0.675–0.936), CR values surpassed the 0.70 cutoff (range: 0.81–0.94), and AVE values were all above the 0.50 threshold (range: 0.51–0.84), collectively supporting convergent validity. Discriminant validity was evaluated by comparing the square root of the AVE for each construct with the inter-construct correlations, and all AVE square roots were greater than their corresponding off-diagonal correlations, confirming that each construct was empirically distinct (detailed CFA results are provided in Supplementary Tables S1, S2).

### 3.3 Descriptive statistics and correlational analysis

Descriptive statistics revealed that students generally perceived the motivational climate in PE classes positively, with mean scores for both mastery and PCs exceeding 3. The levels of basic psychological need satisfaction were favorable, with mean scores of 5.29 for autonomy, 5.58 for competence, and 5.91 for relatedness. The mean score for intrinsic motivation in PE learning was 5.38. The mean for class engagement was 6.19, indicating a relatively high overall level of participation. The skewness and kurtosis values for all variables fell within acceptable ranges (skewness: −1.404 to −1.322; kurtosis: −0.679 to 2.015). Examination of P–P and Q-Q plots confirmed that the data approximately followed a normal distribution, thus satisfying the assumptions for parametric tests.

Partial correlation analysis, controlling for gender, age, and grade, was conducted (see Table 1). The results showed that all dimensions of the main variables were significantly and positively correlated with each other (0.177 ≤ r ≤ 0.741, *p* < 0.001). These significant bivariate relationships provide a foundation for the subsequent mediation effect analysis.

**Table 1:** Correlation analysis among variables.

Subscale	1	2	3	4	5	6	7
1. MC	(0.84)						
2. PC	0.418***	(0.73)					
3. Autonomy	0.45***	0.482**	(0.87)				
4. Relatedness	0.514***	0.501***	0.741***	(0.91)			
5. Competence	0.44***	0.372***	0.648***	0.632***	(0.88)		
6. Intrinsic motivation	0.25***	0.177***	0.368***	0.343***	0.285***	(0.86)	
7. Engagement	0.57***	0.365***	0.549***	0.615***	0.537***	0.384***	(0.83)
M	4.49	3.60	5.26	5.90	5.56	5.37	6.19
SD	0.60	0.91	1.41	1.17	1.29	1.51	1.00

Cronbach’s alpha coefficients are provided along the diagonal; M, mean; SD, standard deviation; Bivariate correlations among the study variables are significant at the *p* < 0.001 level. *** *p* < 0.001.

### 3.4 Hypothesis testing results

To account for potential confounding effects, gender, age, and grade were entered as control variables in the structural model, predicting all endogenous variables. The inclusion of these covariates did not substantially change the path coefficients or significance levels reported below; hence, the results are presented without covariates for parsimony. The hypothesized structural equation model was tested using the maximum likelihood estimation method. The model fit indices were as follows: *χ^2^/df* = 5.43, GFI = 0.911, CFI = 0.924, NFI = 0.906, RMSEA = 0.062. All indices met the recommended standards for acceptable fit, indicating the model was suitable for subsequent path and mediation effect analyses. The standardized path coefficients and hypothesis testing results for the final model are presented in detail in Table 2, with the path relationships visualized in Figure 2.

**Table 2:** Results of hypothesis testing.

Label	Path	Estimate	S. E.	*p*-value	Result
H1a	MAS → AUT	0.331***	0.085	<0.001	Supported
H1b	MAS → REL	0.389***	0.070	<0.001	Supported
H1c	MAS → COM	0.389***	0.068	<0.001	Supported
H2a	PER → AUT	0.538***	0.049	<0.001	Not supported
H2b	PER → REL	0.369***	0.063	<0.001	Not supported
H2c	PER → COM	0.494***	0.047	<0.001	Not supported
H3a	AUT → INTR	0.273**	0.039	0.003	Supported
H3b	REL → INTR	0.042(n.s.)	0.045	0.35	Not supported
H3c	COM → INTR	0.152***	0.046	<0.001	Supported
H4	INTR → ENG	0.207***	0.015	<0.001	Supported

MAS, Mastery climate; PER, Performance climate; AUT, Need for autonomy; REL, Need for relatedness; COM, Need for competence; INTR, Intrinsic motivation; ENG, PE engagement. ***p< 0.001, **p< 0.01, *p<0.05. “n.s.” denotes not significant. The same abbreviations apply hereinafter.

**Figure 2:**
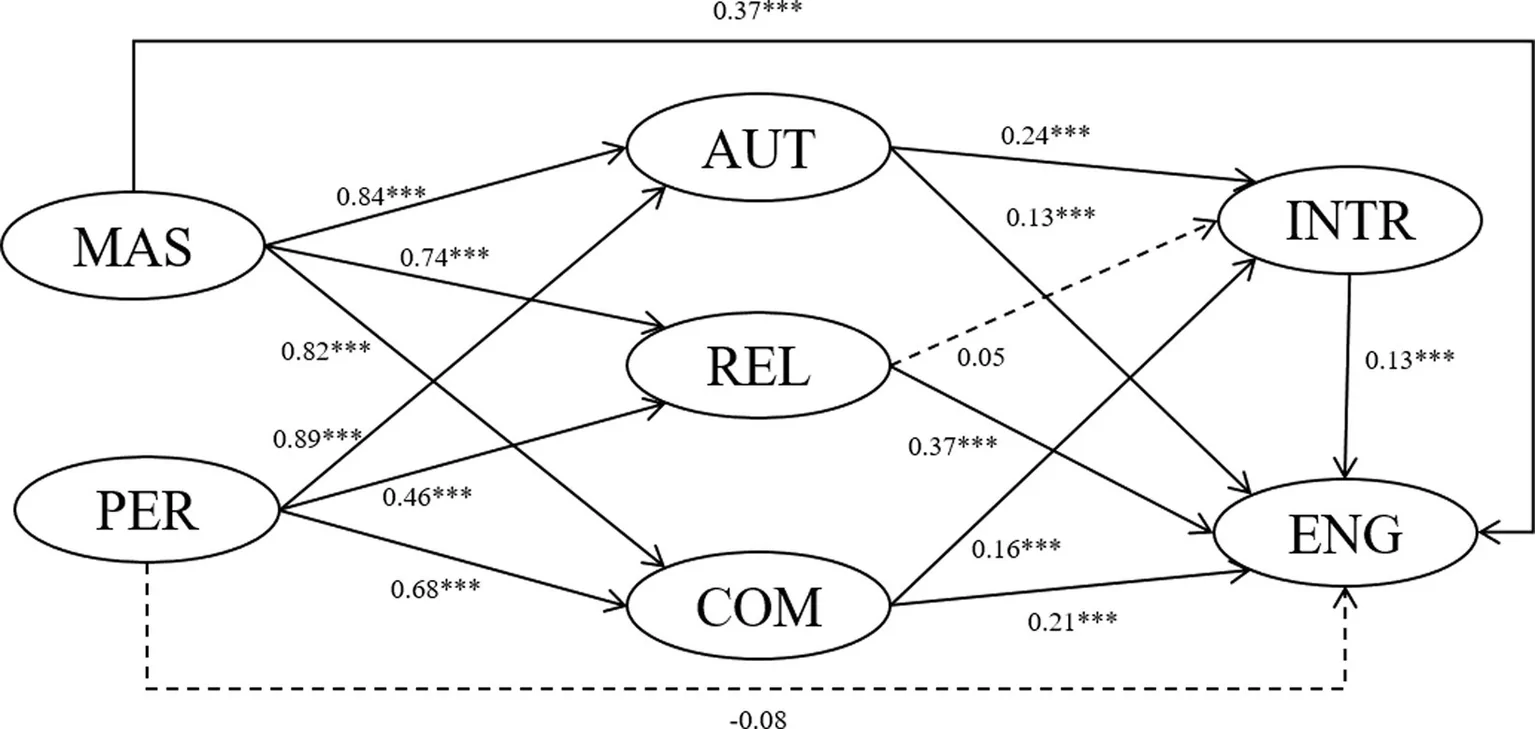
Standardized beta estimates from structural equation modelling. The dashed line represents a non-significant result.

Regarding the predictive effects of the PE class motivational climate on basic psychological needs, the MC demonstrated significant positive predictive effects on autonomy need, relatedness need, and competence need (all *p* < 0.001). Therefore, hypotheses H1a, H1b, and H1c were all supported. Contrary to the hypothesized negative effects, the path coefficients from the PC to autonomy need, relatedness need, and competence need were all significantly positive. Consequently, hypotheses H2a, H2b, and H2c were not supported.

In the test of the predictive effects of basic psychological needs on intrinsic motivation, autonomy need had a significant positive predictive effect on intrinsic motivation (*p* < 0.01), supporting H3a. Competence also showed a significant positive predictive effect on intrinsic motivation (*p* < 0.05), supporting H3c. However, the predictive path from relatedness need to intrinsic motivation was not statistically significant; thus, H3b was not supported.

Finally, in testing the predictive effect of intrinsic motivation on engagement, intrinsic motivation exhibited a significant positive predictive effect on student engagement (*p* < 0.001), providing support for H4.

We further employed the bootstrap procedure with 5,000 resamples to test the indirect effects, with 95% bias-corrected confidence intervals reported in Table 3. For MC, the total and direct effects on engagement were significant (B = 0.763, 95% CI [0.047, 0.994]; B = 0.374, 95% CI [0.270, 1.248]), whereas the indirect effect was not (B = 0.388, 95% CI [−1.085, 0.562]), indicating no mediation; thus, H5a was not supported. For PC, the total effect was significant (B = 0.260, 95% CI [0.123, 0.938]), the direct effect was negative and small, with the 95% CI just excluding zero (B = −0.081, 95% CI [−1.824, −0.006]), and the indirect effect was significant and positive (B = 0.340, 95% CI [0.162, 2.636]), indicating a significant mediation contrary to the primary AGT prediction; therefore, H5b was not confirmed. For autonomy need, the total, direct, and indirect effects on engagement were all significant (indirect effect = 0.031, 95% CI [0.014, 0.054]), indicating a significant mediation effect; thus, H6a was supported. For relatedness need, the total and direct effects were significant, but the indirect effect was not (B = 0.006, 95% CI [−0.008, 0.022]), indicating no significant mediation; therefore, H6b was not supported. For competence need, the total, direct, and indirect effects were all significant (indirect effect = 0.021, 95% CI [0.0004, 0.042]), indicating a significant mediation effect; thus, H6c was supported.

**Table 3:** Mediation effect analysis.

Path	Total Effect		Direct Effect		Indirect Effect	
	Effect	95% CI	Effect	95% CI	Effect	95% CI
MAS → ENG	0.763*	[0.047, 0.994]	0.374**	[0.270, 1.248]	0.388	[−1.085, 0.562]
PER → ENG	0.260**	[0.123, 0.938]	−0.081*	[−1.824, −0.006]	0.340**	[0.162, 2.636]
AUT → ENG	0.162**	[0.095, 0.495]	0.131**	[0.065, 0.469]	0.031**	[0.014, 0.054]
REL → ENG	0.085*	[0.017, 0.203]	0.079*	[0.016, 0.194]	0.006	[−0.008, 0.022]
COM → ENG	0.230**	[0.163, 0.425]	0.210**	[0.144, 0.408]	0.021*	[0.0004, 0.042]

Effect = unstandardized estimate (B). Bootstrap = 5,000 resamples; 95% bias-corrected confidence intervals were obtained from the same bootstrap run. Significance is based on bootstrap two-tailed p values: *p < 0.05, **p < 0.01.

## 4 Discussion

### 4.1 Direct effects among variables

This study examined the direct effects of the PE class motivational climate, basic psychological needs, intrinsic motivation, and student engagement using structural equation modeling. The hypothesized model showed acceptable fit, although the *χ^2^/df* ratio and incremental fit indices were not optimal (see Limitations); all estimated paths therefore represent cross-sectional statistical associations rather than verified causal effects.

#### 4.1.1 Direct effects of the PE class motivational climate on basic psychological needs

The test of direct effects revealed that an MC had a significant positive predictive effect on autonomy, relatedness, and competence needs (supporting H1a, H1b, and H1c), a pattern highly consistent with the majority of existing research. From the integrated AGT–SDT perspective, a mastery-oriented climate—emphasizing effort, individual improvement, and cooperative learning—creates a safe and inclusive learning environment that reduces evaluation pressure and competitive anxiety (Ames, 1992). In PE practice, an MC is typically fostered through formative assessment, individualized guidance, and student choice of activities and practice methods, affording students a sense of control and thus satisfying autonomy (Standage et al., 2005); it also promotes peer cooperation and mutual support, satisfying relatedness (Guo et al., 2022); and by focusing on individual progress rather than ability comparison, it helps students develop positive competence perceptions. Contrary to the hypothesized negative effects, the path coefficients from a PC to autonomy, relatedness, and competence needs were all significantly positive, diverging from H2a–H2c and from some traditional AGT research, yet aligning with several recent empirical studies. Traditional AGT suggests that a performance-oriented climate, centered on ability comparison and competitive evaluation, increases competitive pressure and anxiety, limits autonomous choices, and consequently inhibits need satisfaction (Dweck and Leggett, 1988); however, recent work indicates that the effects of a PC are not uniformly negative (Gråstén and Watt, 2017; Huhtiniemi et al., 2022) and may be moderated by cultural context, student characteristics, and class implementation methods. In the Chinese PE context, students accustomed to a competitive educational environment may adapt to competition and transform it into motivation for skill improvement, thereby feeling enhanced competence (Hu et al., 2025); moreover, the PC perceived in this study likely reflected structured competition (e.g., group competitions, skill challenges) rather than pure “malignant competition,” which may promote peer learning and interaction and thus satisfy relatedness, consistent with Rodrigues et al. (2020). Thus, a PC is not invariably need-thwarting in the Chinese PE context; a well-structured performance-oriented approach may coexist with need satisfaction, enriching the cross-cultural application of AGT. Importantly, however, the present measure could not distinguish constructive from destructive competition, and poorly implemented performance climates (e.g., public ability rankings, normative punishment) remain a documented risk for need thwarting and disaffection (Bartholomew et al., 2011).

#### 4.1.2 Direct effects of basic psychological needs on intrinsic motivation

The direct effects test showed that autonomy and competence needs had significant positive predictive effects on intrinsic motivation (supporting H3a and H3c), while the predictive path from relatedness need to intrinsic motivation was not significant (H3b not supported). This pattern largely aligns with the core tenets of SDT, which hold that satisfaction of the three basic psychological needs is the core prerequisite for the formation and maintenance of intrinsic motivation (Deci and Ryan, 2000). Satisfaction of the autonomy need implies that students have the right to make choices in PE learning, and this sense of control allows students to perceive PE as autonomous and enjoyable, thereby stimulating intrinsic motivation (Ryan and Deci, 2017); accordingly, Cheon et al. (2018) found that autonomy support enhances autonomy need satisfaction, which in turn strengthens intrinsic motivation, consistent with this study’s results. Similarly, satisfaction of the competence need involves a positive perception of one’s capabilities: as students gradually master motor skills and complete tasks, they experience a growing sense of competence that boosts their confidence in PE learning and stimulates intrinsic motivation (Standage et al., 2005).

Notably, the non-significant predictive path from relatedness to intrinsic motivation differs from some existing research. Traditional SDT research suggests that satisfying the need for relatedness enhances feelings of belonging and acceptance, thereby promoting intrinsic motivation (Deci and Ryan, 2000). However, in PE, whose core objective is to improve students’ motor skills and physical fitness, student attention may focus more on skill practice and ability improvement than on emotional connections with teachers and peers, potentially weakening the direct influence of relatedness on intrinsic motivation. Furthermore, the effect of relatedness on intrinsic motivation may involve mediation or moderation rather than a direct effect: Kaefer and Chiviacowsky (2021), for example, found that relatedness satisfaction indirectly influences intrinsic motivation by increasing students’ identification and perceived personal value. Therefore, PE teaching should also optimize how relatedness needs are met—by strengthening teacher-student interaction and promoting peer support—so that relatedness satisfaction can genuinely support the fostering of intrinsic motivation.

#### 4.1.3 Direct effect of intrinsic motivation on engagement

The test of direct effects showed that intrinsic motivation had a significant positive predictive effect on student engagement (H4 supported by data), aligning closely with SDT’s theoretical expectations and a substantial body of empirical research. Pelletier et al. (2001) indicated that students with higher intrinsic motivation are more likely to engage in physical activity long-term and demonstrate greater initiative and persistence in participation, consistent with this study’s findings. When students develop an intrinsic interest in PE, they voluntarily invest more time and effort in physical activities, enhancing the quality of class participation and potentially contributing to lifelong exercise habits.

### 4.2 Analysis of mediating effects

To further reveal the underlying mechanisms between the PE class motivational climate, basic psychological needs, intrinsic motivation, and engagement, this study examined the chain-mediating role of basic psychological needs and intrinsic motivation, as well as the separate mediating role of intrinsic motivation between basic psychological needs and engagement.

#### 4.2.1 Chain-mediating effect of basic psychological needs and intrinsic motivation

Neither hypothesis H5a nor H5b was supported by the data, and both results warrant in-depth analysis considering theoretical logic and practical context.

Chain Mediation under an MC: The test results showed that the total and direct effects of an MC on engagement were significant, while the indirect effect was B = 0.388, with a 95% confidence interval [−1.085, 0.562] that included zero. This indicates that the chain-mediating effect of basic psychological need satisfaction and intrinsic motivation was not significant; thus, H5a was not supported. This suggests that an MC primarily influences student engagement through a direct pathway, rather than via the chain “MC → basic psychological needs → intrinsic motivation → engagement.” This finding shows some divergence from the motivational sequence model of “social context → psychological needs → intrinsic motivation → behavioral outcomes” proposed by Standage et al. (2005). An MC itself possesses strong direct motivational properties that can immediately influence student participation. By emphasizing effort, improvement, and cooperation, it creates a positive, inclusive class environment that reduces student anxiety and psychological pressure, making students more willing to participate actively. This direct environmental incentive might outweigh the indirect effect of the chain mediation, leading to a non-significant chain-mediating effect. Research by Grasten and Watt (2016) also found that a mastery-oriented class climate directly enhances student engagement, with its direct effect being more significant than indirect effects, consistent with this study’s results. A further statistical consideration is that the total indirect effect aggregates multiple specific indirect pathways (through autonomy, relatedness, and competence needs) that may operate in opposite directions and cancel one another out; the wide confidence interval of the MC indirect effect [−1.085, 0.562] is consistent with such suppression, and future research should decompose the specific indirect effects rather than interpret the aggregate alone. Similar null sequential chains have been reported for Chinese secondary PE samples (Chen et al., 2020), suggesting that the direct contextual effect of an MC is not always fully transmitted through the classic motivational sequence. Furthermore, individual differences in the sample (e.g., developmental stage of adolescents, varying levels of pre-existing intrinsic motivation) might have influenced the manifestation of the chain-mediating effect.

Chain Mediation under a PC: The total effect was B = 0.260 [95% CI (0.123, 0.938)], and the direct effect was B = −0.081 [95% CI (−1.824, −0.006)]. The indirect effect was significant whereas the direct effect was not, and the direction of the indirect effect was positive, contrary to the primary AGT prediction. Therefore, H5b was not confirmed. This is a significant finding of this study, showing considerable divergence from traditional AGT expectations. This positive chain-mediating effect is closely related to the earlier finding that a PC positively predicts basic psychological needs. The positive prediction of autonomy, relatedness, and competence needs by a PC established the foundation for this positive mediation. In the present data, a PC was associated with greater satisfaction of the three basic psychological needs, which in turn was associated with higher intrinsic motivation and greater class engagement, forming a positive sequential association: “PC → basic psychological needs → intrinsic motivation → engagement.” This contrasts with the traditional AGT view of a PC’s negative effects, enriching research on its mechanisms. In the collectivist cultural context of China, where students may value group honor and self-improvement, the competitive pressure from a PC might be transformed into motivation for skill enhancement (Hu et al., 2025). PCs in PE, often manifested as group competitions or skill assessments, can stimulate students’ competitive spirit, allowing them to feel improved competence through competition, thereby satisfying their competence need. Existing research also supports this result. Rodrigues et al. (2020) found that a PC created by peers positively influences students’ basic psychological needs, thereby enhancing their motivation and participation levels. The positive chain-mediating effect of a PC in this study reflects its unique mechanism within the Chinese PE context, offering a new perspective on the functioning of a PC within the Chinese PE context. The non-significant direct effect suggests that a PC influences engagement primarily indirectly through psychological mechanisms. Pending experimental verification, these correlational findings suggest that if teachers introduce competitive elements, they should structure them so that challenge is balanced with student experience and skill development.

#### 4.2.2 Separate mediating effect of intrinsic motivation between basic psychological needs and engagement

Among the H6 series hypotheses, H6a and H6c were supported, while H6b was not, reflecting the differential impact paths of the three needs. SDT posits that satisfaction of the autonomy need is a core prerequisite for intrinsic motivation: when individuals feel a sense of autonomy, they are more willing to engage voluntarily, leading to higher behavioral engagement (Deci and Ryan, 2000). In PE, autonomy satisfaction allows students to choose, fostering a sense of control and enjoyment that stimulates intrinsic motivation; accordingly, Hein and Jõesaar (2015) found that autonomy support enhances engagement by satisfying autonomy needs and stimulating intrinsic motivation. Satisfaction of the competence need involves a positive perception of one’s capabilities: in PE, mastering skills and completing tasks provides a sense of competence, directly boosting confidence and initiative in participation and thereby enhancing engagement (Standage et al., 2005); this sense of competence can also stimulate intrinsic motivation, as perceived self-improvement sparks interest in PE—forming the “competence need → intrinsic motivation → engagement” path (Ntoumanis, 2001). While the total and direct effects of relatedness on engagement were significant, the indirect effect’s 95% confidence interval [−0.008, 0.022] included zero, indicating no significant mediating role for intrinsic motivation. This echoes the H3b result, further suggesting that relatedness influences engagement primarily directly, possibly through other mediators. Therefore, teachers should focus on fostering teacher-student interaction and peer support to create a harmonious class atmosphere that enhances belonging, which could also help translate relatedness into intrinsic motivation.

In summary, the mediating effects show clear differentiation: an MC primarily affects engagement directly, with non-significant chain mediation; a PC affects engagement mainly through a positive chain-mediating effect, with a non-significant direct effect; and intrinsic motivation shows significant indirect effects for autonomy and competence needs, but not for relatedness need. These findings enrich the application of AGT and SDT in PE. In drawing practical implications, a clear distinction is needed between conclusions supported by the present data and suggestions that remain speculative. The data-supported conclusions are that students perceiving a stronger MC, and those reporting greater autonomy and competence need satisfaction, also report higher intrinsic motivation and engagement, and that intrinsic motivation significantly mediates the associations of autonomy and competence with engagement. By contrast, recommendations to deliberately combine mastery and performance-oriented climates go beyond the present evidence: this was a cross-sectional correlational study without experimental manipulation, and moderators such as gender, prior sport experience, and teacher characteristics were not tested; the positive associations of the PC should therefore not be read as evidence that promoting competition per se will increase engagement. Any introduction of competitive elements should be criterion-referenced and carefully structured, since poorly implemented performance climates can thwart needs and undermine motivation (Bartholomew et al., 2011).

### 4.3 Limitations and future directions

Several limitations should be acknowledged. The cross-sectional design precludes causal inference; all estimated paths reflect statistical associations evaluated under theory-driven assumptions, and bidirectional or reciprocal relationships remain plausible (e.g., students with higher PE engagement may subsequently perceive a more favorable motivational climate and report greater need satisfaction). Methodologically, the model fit was suboptimal (*χ*^2^/df = 5.43, CFI = 0.924, NFI = 0.906), and the nested school structure was not modeled due to the limited number of schools, which may have affected the precision of parameter estimates (Maas and Hox, 2005). In terms of measurement, the sample was confined to two provinces and grades 7–9, limiting generalizability; the intrinsic motivation subscale contained only three items, and the performance climate measure could not distinguish constructive from destructive competition, leaving the mechanisms underlying its positive effects unclear. Regarding data sources, all constructs were assessed via student self-report; although the unmeasured latent method factor analysis suggested that common method variance was not a serious threat, single-source data may still have inflated the observed correlations and path coefficients (Podsakoff et al., 2003).

Future research should employ longitudinal or experimental designs to establish temporal order, expand the sample to diverse regions and grade levels, and incorporate multilevel SEM with a larger school sample. Objective measures alongside self-reports would help reduce common method bias. Examining potential moderators (e.g., gender, prior experience) would further clarify for whom the effects are most pronounced, and qualitative methods could illuminate how Chinese students perceive performance climate in PE. Future studies may also consider using a longer version of the intrinsic motivation measure to enhance construct representation. Translating these findings into targeted interventions remains a critical next step for enhancing student engagement and health outcomes.

## 5 Conclusion

Utilizing structural equation modeling and bootstrap mediation analysis, this study systematically explored the relationships among the PE class motivational climate, basic psychological needs, intrinsic motivation, and student engagement. The core conclusions are as follows: First, the proposed model showed acceptable fit, and significant direct relationships were identified among the variables. An MC positively predicted the satisfaction of all three basic psychological needs (autonomy, relatedness, and competence). Contrary to some theoretical expectations, a PC also positively predicted the satisfaction of all three needs. Autonomy and competence needs positively predicted intrinsic motivation, which in turn positively predicted student engagement. Second, the mediating effects showed distinct patterns. There was no significant chain-mediating effect of basic psychological needs and intrinsic motivation between an MC and engagement. However, a PC exerted a positive indirect effect on engagement through the sequential pathway of basic psychological needs and intrinsic motivation. Furthermore, the indirect effects of autonomy and competence needs on engagement through intrinsic motivation were significant, whereas that of relatedness need was not. The study supports several hypothesized associations and illuminates the pathways linking these key variables, providing a cross-sectional empirical basis—rather than intervention evidence—for discussions on optimizing teaching practices in PE.

## Data Availability

The data analyzed in this study is subject to the following licenses/restrictions: the datasets of this study are available from the corresponding author on reasonable request. Requests to access these datasets should be directed to coolxq521@jnxy.edu.cn.
