# Supplementary material for: Motivational climate, basic psychological needs, intrinsic motivation, and engagement in Chinese students during physical education classes
Source: Front Psychol. 2026 Oct 5;17:1913633. doi: 10.3389/fpsyg.2026.1913633 (PMC13677687; doi:10.3389/fpsyg.2026.1913633)
Supplement: Supplementary file 1 [file Table_1.DOCX]

| Table S1. Standardized Factor Loadings, Composite Reliability, and Average Variance Extracted | | | | |
| --- | --- | --- | --- | --- |
| Construct | Item | Standardized loading | CR | AVE |
| Mastery Climate |  |  | 0.85 | 0.587 |
|  | MC1 | 0.831 |  |  |
|  | MC2 | 0.791 |  |  |
|  | MC3 | 0.747 |  |  |
|  | MC10 | 0.687 |  |  |
| Performance Climate |  |  | 0.807 | 0.512 |
|  | CC8 | 0.772 |  |  |
|  | MC5 | 0.729 |  |  |
|  | MC14 | 0.683 |  |  |
|  | MC16 | 0.675 |  |  |
| Autonomy Need |  |  | 0.868 | 0.622 |
|  | BPN9 | 0.858 |  |  |
|  | BPN3 | 0.769 |  |  |
|  | BPN12 | 0.771 |  |  |
|  | BPN6 | 0.753 |  |  |
| Relatedness Need |  |  | 0.88 | 0.648 |
|  | BPN8 | 0.772 |  |  |
|  | BPN2 | 0.758 |  |  |
|  | BPN5 | 0.852 |  |  |
|  | BPN11 | 0.834 |  |  |
| Competence Need |  |  | 0.914 | 0.728 |
|  | BPN4 | 0.861 |  |  |
|  | BPN1 | 0.777 |  |  |
|  | BPN7 | 0.903 |  |  |
|  | BPN10 | 0.866 |  |  |
| Intrinsic Motivation |  |  | 0.843 | 0.642 |
|  | MPE14 | 0.815 |  |  |
|  | MPE5 | 0.797 |  |  |
|  | MPE10 | 0.792 |  |  |
| Engagement |  |  | 0.94 | 0.839 |
|  | PA1 | 0.898 |  |  |
|  | PA2 | 0.914 |  |  |
|  | PA3 | 0.936 |  |  |
| Note: CR = Composite Reliability; AVE = Average Variance Extracted. All standardized factor loadings exceeded .50. CR values exceeded .70 and AVE values exceeded .50 for all constructs, demonstrating adequate convergent validity. | | | | |
