# Supplementary material for: Motivational climate, basic psychological needs, intrinsic motivation, and engagement in Chinese students during physical education classes
Source: Front Psychol. 2026 Oct 5;17:1913633. doi: 10.3389/fpsyg.2026.1913633 (PMC13677687; doi:10.3389/fpsyg.2026.1913633)
Supplement: Supplementary file 2 [file Table_2.DOCX]

| **Table S2. Discriminant Validity** | | | | | | | |
| --- | --- | --- | --- | --- | --- | --- | --- |
| Construct | 1 | 2 | 3 | 4 | 5 | 6 | 7 |
| 1. Mastery Climate | **0.766** |  |  |  |  |  |  |
| 2. Performance Climate | 0.508 | **0.716** |  |  |  |  |  |
| 3. Autonomy Need | 0.538 | 0.569 | **0.789** |  |  |  |  |
| 4. Relatedness Need | 0.518 | 0.425 | 0.733 | **0.805** |  |  |  |
| 5. Competence Need | 0.587 | 0.566 | 0.829 | 0.692 | **0.853** |  |  |
| 6. Intrinsic Motivation | 0.307 | 0.205 | 0.421 | 0.323 | 0.387 | **0.801** |  |
| 7. Engagement | 0.652 | 0.457 | 0.74 | 0.64 | 0.752 | 0.511 | **0.916** |
| Note: Bold values on the diagonal represent the square root of the AVE for each construct. Off-diagonal values are Pearson correlation coefficients between constructs. | | | | | | | |
